# Influence of postoperative D-dimer evaluation and intraoperative use of intermittent pneumatic vein compression (IPC) on detection and development of perioperative venous thromboembolism in brain tumor surgery

**DOI:** 10.1007/s00701-024-06379-2

**Published:** 2024-11-26

**Authors:** Katharina Zimmer, Maximilian Scheer, Christian Scheller, Sandra Leisz, Christian Strauss, Bettina-Maria Taute, Martin Mühlenweg, Julian Prell, Sebastian Simmermacher, Stefan Rampp

**Affiliations:** 1 Department of Neurosurgery University Hospital Halle (Saale), Martin-Luther-University Halle-Wittenberg, Halle, Germany; 2 Department of Neurosurgery, University Hospital Erlangen, Erlangen, Germany; 3 Department of Cardiology, Angiology and Intensive Care Medicine, University Hospital Halle (Saale), Halle, Germany

**Keywords:** Intermittent pneumatic compression, D-dimer, Deep venous thrombosis, Craniotomy, Pulmonary embolism

## Abstract

**Background Objective:**

Venous thromboembolism (VTE), which includes deep vein thrombosis (DVT) and pulmonary embolism (PE), is a common complication in craniotomy patients and is associated with increased morbidity and mortality. The duration of surgery is a known risk factor. Other factors such as positioning and tumor entity have hardly been investigated or are controversial.

In two pilot studies, the determination of plasma D-dimer concentration led to a high detection rate of DVT, while the use of intermittent pneumatic venous compression (IPC) drastically reduced the incidence of VTE. In the present study we investigated the efficacy of the two approaches, either alone or in combination, in a large patient cohort.

**Methods:**

1759 patients who underwent elective craniotomy between 2009 and 2023 were retrospectively analyzed. The staggered use of D-dimer determination and intraoperative use of IPC resulted in 3 groups: Group 1: no procedure; Group 2: D-dimer evaluation; Group 3: IPC and D-dimer evaluation. If the D-dimer level was ≥ 2 mg/l (Fibrinogen equivalent units; FEU), venous ultrasound was performed. Age, gender, tumor entity, duration and extent of surgery, patient positioning, type of VTE were also recorded and analyzed.

**Results:**

The introduction of postoperative D-dimer evaluation increased the rate of detection of thrombosis from 1.7% in group 1 to 22.6% in group 2. The addition of IPC reduced the rate of thrombosis to 4.4%. Age, gender and patient positioning did not affect the rate of VTE. We were able to confirm the duration of surgery as an individual risk factor and showed that WHO grade 4 tumors and metastasis have an increased VTE risk.

**Conclusions:**

If D-Dimer levels are not analyzed routinely about 20% of craniotomy patients suffer from a clinically silent thrombosis. Each with the risk of fate PE. Intraoperative use of IPC during craniotomy dramatically reduces the risk of VTE.

## Introduction

Resection of a brain tumor by craniotomy is a common neurosurgical procedure. A serious complication that occurs in up to 50% of craniotomy patients is venous thromboembolism (VTE), which includes deep vein thrombosis (DVT) and pulmonary embolism (PE) and is associated with significant morbidity and mortality [8, 20]. In the majority of cases, this condition is asymptomatic. However, symptomatic VTE occurs in 7.5% of patients undergoing craniotomy [1, 6, 56] and even asymptomatic isolated distal deep vein thrombosis (IDDVT) localized to soleus and / or gastrocnemius muscle veins can progress to symptomatic deep vein thrombosis [19, 29, 34, 42]. This can lead directly and without clinical warning to pulmonary embolism in 7% of affected patients [13]. Such a pulmonary embolism is caused by DVT of the legs in > 90% of cases. [7], occurs in 3.7% of all patients undergoing craniotomy and is fatal in up to 50% of all affected neurosurgical patients [16, 20].

Several factors may contribute to the high incidence of VTE, including prolonged duration of surgery and mechanical ventilation [22, 37], diabetes mellitus (DM) and new-onset postoperative motor deficits [18], advanced age and high body mass index (BMI) [47]. There is also evidence that asymptomatic, non-ambulatory neurosurgical patients with high-grade cancer may have a higher risk of developing VTE [8]. A link between certain tumor types and the occurrence of VTE is controversial [32, 40, 50].

Early diagnosis of VTE is essential to reduce mortality by initiating treatment in time [55]. In addition to the clinical assessment and pre-test probability (Wells-Score), the determination of the D-dimer level is part of the standard diagnostic procedure for suspected DVT in outpatients. Venous ultrasound investigation is the first-line imaging method for further diagnosis if D-dimers are elevated to detect DVT [21]. If pulmonary embolism is suspected, contrast-enhanced CT of the chest is performed [3, 55]. D-dimer is a small protein fragment present in plasma after a blood clot has been dissolved by fibrinolysis. Elevated plasma levels of D-dimer are used as a diagnostic marker for thrombosis [3, 21]. However, while a negative result rules out thrombosis in most cases, a positive result may indicate thrombosis but does not rule out other potential causes [35]. Surgery is one of the causes of an increase in D-dimer value, which makes postoperative interpretation difficult [21, 55]. In an earlier pilot study of our department, we were able to show that a plasma D-dimer level of ≥ 2 mg/L (FEU) on postoperative day 3 is indicative of VTE with a sensitivity of 95.3% and a specificity of 74.1%, allowing us to define a practical threshold [37].

For DVT prophylaxis, there are two different options: medical treatment with heparin or mechanical prophylaxis with graduated compression stockings (GCS) or intermittent pneumatic venous compression (IPC) [54, 55].

Low molecular weight heparin is widely used for postoperative thromboprophylaxis [27]. However, the number of patients who develop VTE remains high [47]. One problem is that because of the increased risk of bleeding, this therapy is not started intraoperatively, but several hours later [15, 44]. As a result, many patients do not receive adequate thromboprophylaxis during surgery. Consequently, there is an urgent need to improve neurosurgical patient care.

One form of mechanical thrombosis prophylaxis is IPC, which has been used successfully in a number of surgical procedures other than craniotomy [26, 54]. IPC takes advantage of a multi-chambered cushion, which is wrapped around the lower legs and in- and deflated, at intervals during surgery. It is aimed to mimic the compression of the lower- extremity veins by muscle contraction [17]. For craniotomy, a recently published systematic review and meta-analysis of 5 studies indicated that IPC may reduce the incidence of VTE in patients receiving neurosurgical interventions [36]. However, due to the limited number of studies available a strong recommendation could not be given, indicating that additional randomized controlled trials are needed to draw a definite conclusion [36]. In a second pilot study of our department, it was shown in 94 craniotomy patients that intra-operative IPC drastically reduces the risk of VTE: from 26.4% in the control group to 7.3% in the IPC group [38].

Both methods (intra-operative IPC and post-operative D-dimer) are now used on a routine basis in our department. The introduction of postoperative D-dimer determination in 2012 and the intraoperative use of IPC in 2016 resulted in different cohorts in which neither procedure was used, only postoperative D-dimer was determined, or both procedures were combined. In the present study, we sought to determine whether D-dimer determination with or without the additional intraoperative use of IPC has an impact on the incidence and detection of postoperative DVT or PE in craniotomy patients. Data from 1759 patients who underwent elective craniotomy between 2009 and 2023 were retrospectively analyzed.

## Methods

### Data collection and patient population

**Fig. 1:**
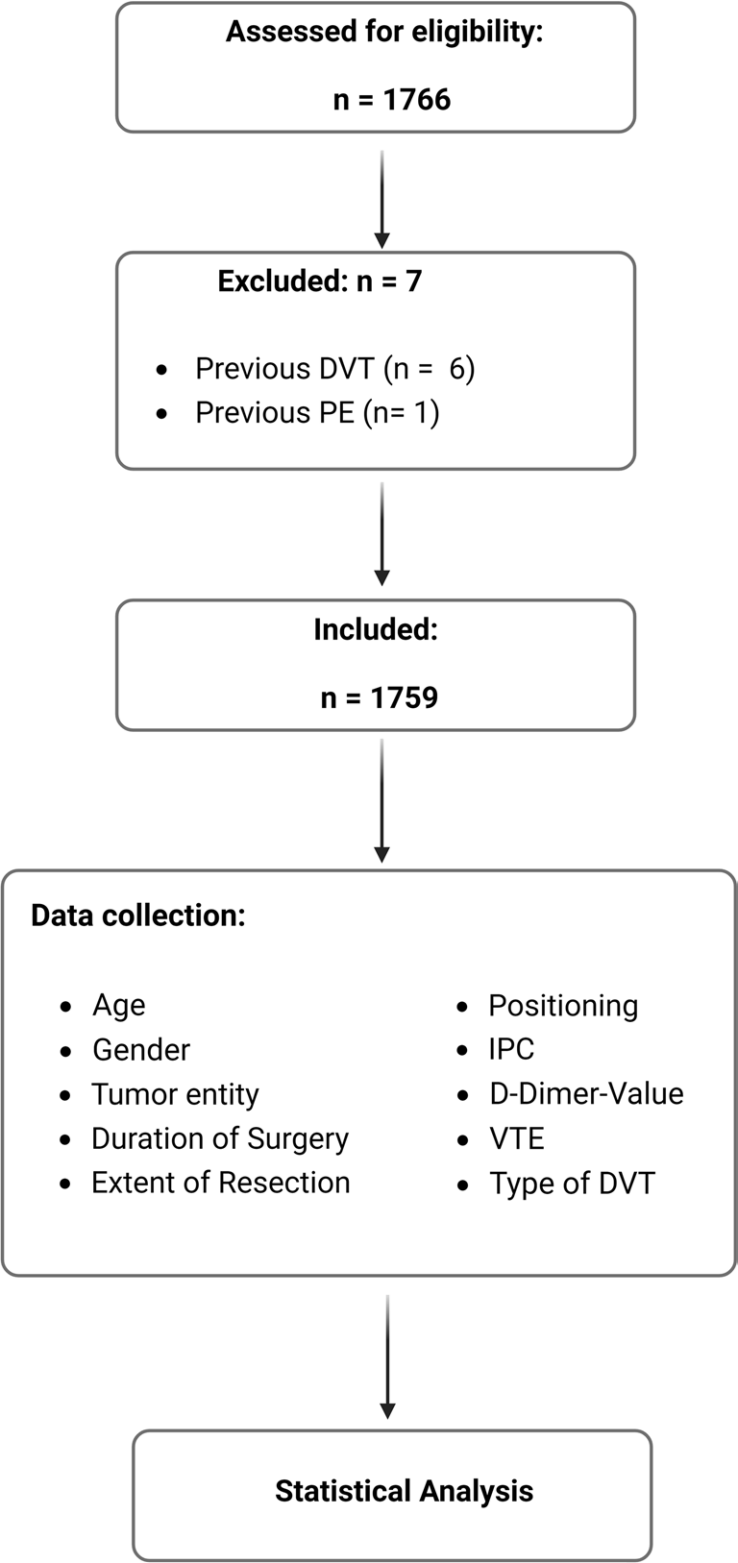
Flow chart of the study

Data of 1759 patients who underwent craniotomy at the Department of Neurosurgery, University Hospital of Halle, Germany, between 2009 and 2023 were anonymized and retrospectively analyzed. Inclusion criteria were craniotomy for suspected brain tumor between 2009 and 2023. Patients who had undergone multiple surgeries due to tumor recurrence were also included. Exclusion criteria were pre-existing thrombosis, PE, or thrombophilia. Of the initial 1766 patients, seven were excluded due to these criteria (Fig. 1).

The parameters recorded and analyzed for our patients were: Age, gender, type of brain tumor, duration and extent of surgery (gross total resection (GTR), subtotal resection (STR), biopsy). The World Health Organization's (WHO) current version of central nervous system tumor classification was used for tumor entity [23–25]. In addition to these entities, metastases were also recorded. If the pathological work-up revealed inflammatory diseases, vascular processes or lymphomas, they were included in the category "other". Patient positioning during surgery was categorized as supine, prone, sitting, or half side position. In addition, we assessed D-dimer levels, the use of IPC during surgery, and whether and what type of thrombosis the patient had: typical lower extremity DVT, muscle vein thrombosis, or thrombosis associated with a central venous catheter (CVC). It was also recorded whether a PE was detected. Figure 1 shows a flow chart of the study.

**Fig. 2:**
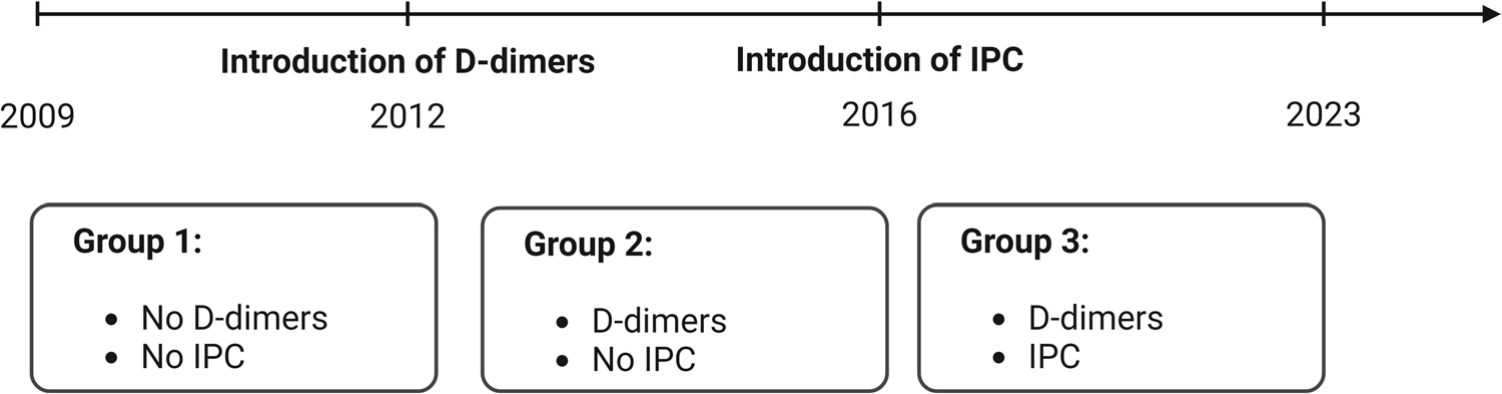
Overview of the timeline of the establishment of the methods of D-dimer determination and application of IPC, with the resulting groups

Patients were divided into three subgroups: In the first group, neither postoperative D-dimers were measured nor intraoperative IPC was applied. In patients in the second group, D-dimer levels were analyzed on the third day after craniotomy, but no IPC was applied during surgery. In the third group, intraoperative IPC was used and D-dimer levels were evaluated on the third postoperative day (Fig. 2).

### Intraoperative IPC Administration

Before positioning the patient, standard calf-length IPC sleeves (multichamber) were applied over the regular compression stockings. Throughout the entire procedure until extubation, these were intermittently inflated to a maximum pressure of 45 mmHg in standard prophylaxis mode using a standard air pulse generator (Kendall SCD 700, Cardinal Health): sequential compression (inflating the distal chambers first, then moving to the more proximal chambers in a wave-like, "milking" fashion) was applied once every minute. The procedure corresponds to the one in our pilot study [38].

### Venous ultrasound investigation and Diagnosis of PE

If D-dimer levels were ≥ 2 mg/L (FEU), these patients underwent Doppler ultrasound. Diagnosis of DVT was based on direct thrombus detection by incomplete compressibility of the vessel and/or observation of absent blood flow, as described previously [38]. If the patient had suspicion of PE, chest CT with contrast was performed.

### Statistical analysis

Baseline data were analyzed using descriptive statistics, including means/standard deviation for rationally scaled data and median/quartiles for ordinally scaled data. Differences between groups were evaluated with ANOVA, and chi-square tests. P-values below 0.05 were considered statistically significant. Influence of gender, age, type of surgery, patient positioning, duration of surgery, IPC and D-Dimer determination on the probability of a thrombosis were investigated using linear regression analysis. All analysis steps were conducted using R 4.1.2 [41].

## Results

### Patient characteristics

Of the 1759 patients, 966 (54.3%) were female with a mean age of 54.9 years. There were slight differences in age between the groups, with a value of 55.4 ± 18.6 years in group 1, 56.0 ± 14.5 in group 2, and 53.5 ± 18.3 in the last group (p = 0.04). All groups had more female patients. The following values were found for the individual groups: The first group included 289 (49.6%) women, group 2 397 (56.6%) and the last group 269 (56.8%) (p = 0.02). In all groups, benign tumors (WHO grade 1) were the most common, with 276 (47.3%) in group 1, 384 (54.7%) in group 2, and 261 (55.1%) in group 3. Nearly one-third of patients in each group had a WHO grade 4 tumor or metastatic disease. In detail for WHO grade 4: 106 (18.2%) in group 1; 125 (17.8%) in group 2; 61 (12.9%) in group 3. For metastases, the following values were found: 82 (14.1%) in group 1; 88 (12.5%) in group 2; and 84 (17.7%) in group 3. WHO grade 2 and 3 tumors were found much less frequently. In the first group, 34 patients (5.8%) had a grade 2 tumor, in group 2, 48 patients (6.8%), and in group 3, 24 patients (5.1%). The values for WHO grade 3 tumors were 36 (6.2%) in group 1, 24 (3.4%) in group 2, and 3 (0.6%) in group 3. In the first group, 49 patients (8.4%) underwent surgery for another intracranial pathology (category other), 33 patients (4.7%) in group 2, and 41 patients (8.6%) in group 3. The distribution of entities showed significant differences in Chi-square (p < 0.001). Data are shown in Table 1.

**Table 1:** Baseline data of patients assigned to the three categorized groups

	Group 1	Group 2	Group 3	p value	Test
Number (n)	583	702	474		
Age (mean, SD)	55.4 ± 18.6	56.0 ± 14.5	53.5 ± 18.3	0.04	ANOVA
Gender (Women)	289 (49.6%)	397 (56.6%)	269 (56.8%)	0.02	Chi square
Extent of resection Biopsy STR GTR	80 (13.7%) 75 (12.9%) 428 (73.4%)	44 (6.3%) 80 (11.4%) 577 (82.2%)	19 (3.9%) 84 (17.1%) 387 (78.7%)	< 0.001	Chi square
Duration of Surgery (mean,SD)	229.9 ± 132.1	286.2 ± 126.4	273.3 ± 120.6	< 0.001	ANOVA
Thrombosis	10 (1.7%)	159 (22.6%)	21 (4.4%)	< 0.001	Chi square
Thrombosis type DVT Muscle vein Central venous line	1 (10.0%) 3 (30.0%) 1 (10.0%)	116 (73.0%) 57 (35.8%) 45 (28.3%)	14 (66.7%) 5 (23.8%) 2 (9.5%)	0.003	Chi square
PE	3 (0.5%)	36 (5.1%)	4 (0.8%)	< 0.001	Chi square
Positioning Supine Prone Seated Half side	475 (81.5%) 28 (4.8%) 54 (9.3%) 26 (4.5%)	591 (84.2%) 41 (5.8%) 31 (4.4%) 39 (5.6%)	343 (72.4%) 27 (5.7%) 26 (5.5%) 78 (16.5%)	< 0.001	Chi square
Tumor entities WHO 1 WHO 2 WHO 3 WHO 4 Metastasis Other	276 (47.3%) 34 (5.8%) 36 (6.2%) 106 (18.2%) 82 (14.1%) 49 (8.4%)	384 (54.7%) 48 (6.8%) 24 (3.4%) 125 (17.8%) 88 (12.5%) 33 (4.7%)	261 (55.1%) 24 (5.1%) 3 (0.6%) 61 (12.9%) 84 (17.7%) 41 (8.6%)	< 0.001	Chi square

### Operative details

The following section describes the surgical details such as the duration of the procedure, the extent of the resection, and the positioning of the patient in the three groups created. The duration of surgery in each group varied (mean, SD) between 229 ± 132 min in group 1, 286.2 ± 126.4 in group 2, and 273.3 ± 120.6 min in group 3 (p < 0.001). Regarding the extent of resection, biopsy, STR and GTR were distinguished. GTR was most common in all groups with values of 428 (73.4%) in group 1, 577 (82.2%) in group 2 and 387 (78.7%) in group 3. Biopsies were performed less frequently during the study period and were carried out in 80 patients (13.7%) in group 1, 44 patients (6.3%) in group 2 and 19 patients (3.9%) in group 3. For the extent of resection, the chi-square test showed significant differences between the groups (p < 0.001). The patient's position was also recorded and classified as supine, prone, sitting, and half side. Supine position was the most common in all groups and was found in 475 patients (81.5%) in group 1, 591 patients (84.2%) in group 2, and 343 patients (72.4%) in group 3. The other positions were less common. For the prone position, there were 28 cases (4.8%) in group 1, 41 cases (5.8%) in group 2, and 27 cases (5.7%) in group 3. For the sitting position, there were 54 cases (9.3%) in group 1, 31 cases (4.4%) in group 2, and 26 cases (5.5%) in group 3. For the half side position, we found 26 patients (4.5%) in group 1, 39 patients (5.6%) in group 2, and 78 patients (16.5%) in group 3. For positioning, the chi-square test showed significant differences between the groups (p < 0.001). The data mentioned here is shown in Table 1.

### Incidence of VTE

Both types of VTE, DVT and PE, were recorded for the three groups. The diagnosis of DVT was least frequent in group 1 with a value of 1.7% (10 patients). This increased to 22.6% (159 patients) in the second group when D-dimer was determined. Only 4.4% of patients (21 cases) in Group 3 were found to suffer from DVT when using IPC intraoperatively. These differences were found to be highly significant (p < 0.001) using the Chi square test. We also recorded the type of thrombosis. However, in some cases, which occurred a long time ago, this was no longer traceable. In group one, there was one traceable case of DVT (0.2%), 3 cases of muscle vein thrombosis (0.5%), and 1 case of CVC-associated thrombosis (0.2%). In the second group, some cases occurred with several types of thrombosis simultaneously. There were 116 cases with DVT (16.5%), 57 cases with muscle vein thrombosis (8.1%), and 45 cases with CVC-associated thrombosis (6.4%). In the last group, 14 cases of DVT (2.9%), 5 cases of muscle vein thrombosis (1.1%) and 2 cases of CVC-associated thrombosis (0.4%) were identified. Again, significant differences were found between the groups using the chi-squared test (p = 0.003).

Regarding the detection of PE, the lowest number was found in group 1 with a value of 0.5% (3 cases). In group 2, this rate increased to 5.1% (36 cases). The use of intraoperative IPC resulted in only 4 cases (0.8%) of PE in group 3. For this complication, the differences between the groups were also highly significant (p < 0.001). The chi-square test was used for the assessment.

### Factors associated with VTE

In order to identify the factors that increase the risk of thrombosis, a regression analysis was carried out. The duration of the surgery proved to be a decisive risk factor (p < 0.002). Similarly, surgical treatment without the use of intraoperative IPC was strongly associated with an increased risk of VTE compared to surgery with intraoperative IPC (p = 0.004). Significantly more VTE were detected by postoperative D-dimer measurement (p < 0.0001). The correlation between tumor entity and thrombosis risk was also analyzed. It was found that higher grade tumors of WHO grade 4 (p = 0.035) and metastases (< 0.0001) had an increased risk of VTE. All other factors showed no influence with regard to VTE. Factors such as gender, age, extent of resection and positioning of the patient during the operation were analyzed. Other histologies such as WHO grade 1, grade 2, grade 3 and “other” were also not associated with an increased risk of thrombosis. The data from the regression analysis are shown in Table 2.

**Table 2:** Logistic Regression analysis evaluating the probability of VTE

	**Estimate**	**Error**	**Z-Value**	**p value**
Intercept	−2.626	133.9	−0.020	0.984
Gender (female)	0.06853	0.1764	0.388	0.697
Age	0.01099	0.006113	1.797	0.072
Duration of surgery	0.002883	0.0006824	4.225	** < 0.0002**
Type of surgery	10.20	359.2	0.028	0.977
Patient positioning (prone position) compared to supine position	0.4862	0.4250	0.144	0.252
Patient positioning(seated) compared to supine position	0.04350	0.3868	0.112	0.910
Patient positioning (half side position) compared to supine position	0.2694	0.3632	0.742	0.458
No IPC use compared to IPC use	1.097	0.3894	2.817	**0.004**
D-Dimer determination	2.550	0.3206	7.953	** < 0.0001**
Diagnosis WHO Grade 2 compared to WHO grade 1	3.683	3.419	1.077	0.281
Diagnosis WHO Grade 3 compared to WHO grade 1	−9.015	7.600	−1.186	0.235
Diagnosis WHO Grade 4 compared to WHO grade 1	5.175	2.460	2.104	**0.035**
Diagnosis metastasis compared to WHO grade 1	8.535	2.585	3.302	** < 0.0001**
Diagnosis other compared to WHO grade 1	2.973	4.219	0.705	0.481

**Fig. 3:**
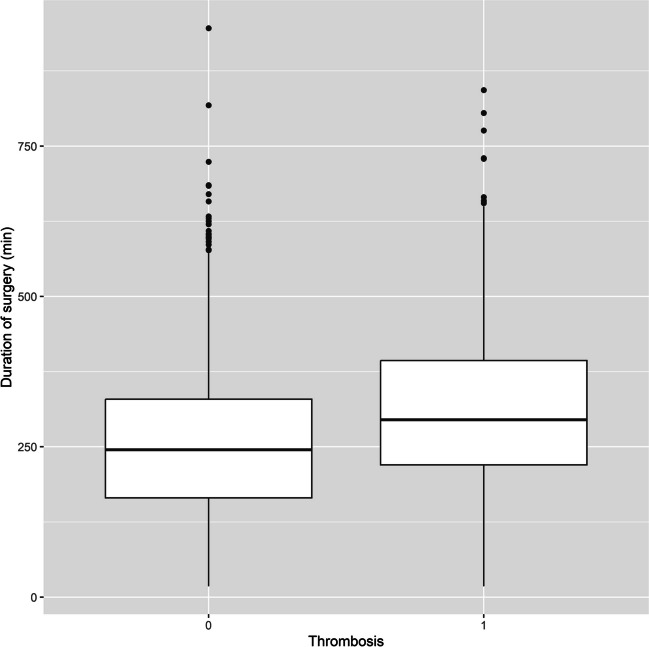
Comparison of the duration of surgery in patients with no evidence of thrombosis (Left) and with evidence of thrombosis (Right) illustrated as Boxplot (1st—3rd quartile). The line represents the median value

Patients without evidence of thrombosis had a shorter operative time than patients with postoperative VTE. The median duration of surgery for the former population was 245 min (1st – 3rd quartil: 165–329 min) without evidence of VTE and 295 min (1st-3rd quartile: 220–393.5 min, p < 0.001) with evidence of VTE (Fig. 3).

**Fig. 4:**
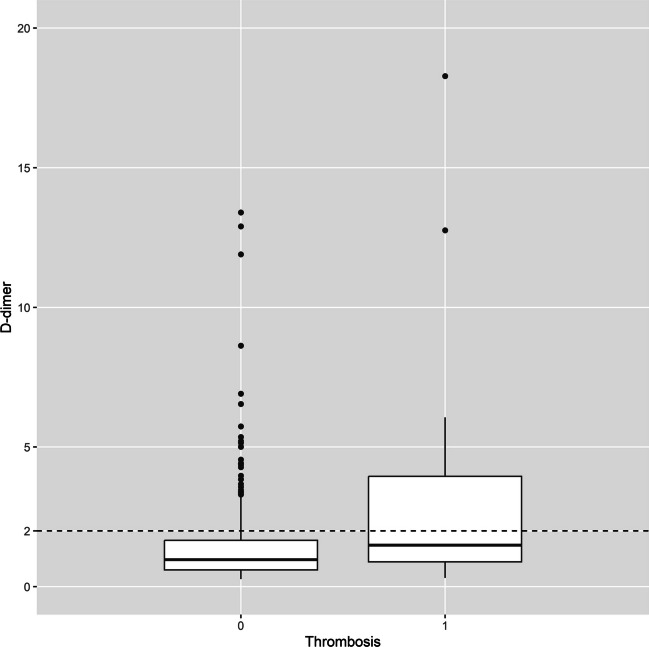
Comparison of D-dimer levels in patients without evidence of thrombosis (left) and with evidence of thrombosis (right) illustrated as Boxplot (1st—3rd quartile). The line represents the median value. The dashed line represents the previous cut-off value of 2 mg/L

We analyzed the data of the patients using intraoperative application regarding the postoperative D-dimer levels and the relationship with the detection of VTE. It was found that patients without thrombosis had a value of 0.97 (median; 1st – 3rd quartile: 0.60 – 1.66) and patients with thrombosis had a value of 1.49 (median; 1st – 3rd quartile: 0.89 – 3.95). The value from the pilot study, in which the IPC was not activated intraoperatively, was 2.0 mg/L. These data are presented in Fig. 4.

## Discussion

Both DVT and PE, collectively referred to as VTE, are common complications in patients undergoing craniotomy. Rates vary widely in the literature, ranging from 3.5% to 47% [14, 22, 51]. In this study, we were able to show that post-operative D-dimer measurement significantly increases the detection rate of potentially asymptomatic thromboses, allowing early treatment and thus reducing the risk of possibly fatal PE. On the other hand, we were able to show that the intraoperative use of IPC, using the same diagnostic algorithm, dramatically reduces the risk of VTE, further improving patient safety.

Compared to other procedures, craniotomy patients are at high risk for VTE. This is due to the relatively long duration of surgery compared to other specialties, the often malignant nature of the underlying disease, and the release of procoagulant factors from the brain tissue [10, 28, 46, 55].

The duration of surgery is a known risk factor, with a significant increase in risk after 4 or 6 h, depending on the literature [22, 48, 49, 51]. In the retrospective analysis presented here, we were also able to confirm the duration of surgery as an individual risk factor, which proved to be highly significant in the regression analysis (p < 0.0002). The average duration of surgery was 245 min in patients without evidence of VTE and 295 mi in patients with evidence of VTE. One factor that is at least partially related to duration of surgery is the extent of resection. However, this was not associated with an increased risk of VTE in our analysis.

One of the standard measures for the diagnosis of VTE is the determination of D-dimer values [55]. However, the use of D-dimer in the postoperative period remains controversial because surgery is known to affect the value [31, 52]. In a pilot study of our department, we demonstrated that a value of 2 mg/L on the third postoperative day was associated with high sensitivity and specificity. However, the study population was relatively small (101 patients) and IPC was not used intraoperatively [37]. In this now significantly larger cohort, the determination of postoperative D-dimer levels was associated with a dramatic increase in the detection of VTE from 1.7% in group 1 to 22.6% in group 2. In contrast to group 1, in which only symptomatic patients received diagnostic testing for VTE, in group 2, diagnostic testing (Venous ultrasound investigation or CT if there was suspicion of PE) was performed if the reference level of 2 mg/L was exceeded or, of course, if the patient had clinical signs of VTE even if the reference level was not exceeded. Another possible reason for the significantly higher number of VTE is the different duration of surgery in these two groups (229.9 min in group 1 vs. 286.2 min in group 2).

Other authors have also investigated the significance of postoperative D-dimer. A cut-off value of 1.5 mg/L on the third postoperative day in patients with brain tumors was considered appropriate [33]. In contrast, other authors were only able to show that D-dimer peaked on the third to fourth postoperative day and that the levels did not differ in patients with or without VTE [30]. Our regression analysis showed that the determination of postoperative D-dimer was significantly associated with the diagnosis of VTE (p < 0.0001), so we consider this a useful tool for the early diagnosis of VTE. In contrast to the pilot study, intraoperative IPC was used in addition to postoperative D-dimer determination. In patients with VTE and intraoperative IPC, a median value of 1.49 mg/L was found, which may suggest that the original reference value may need to be redefined. This question cannot be answered conclusively with this retrospective study. A prospective, randomized study would be required to answer this question.

For VTE prophylaxis, there are two options. One is the administration of heparin derivatives. The other is mechanical prophylaxis, which can be divided into GCS and IPC [11, 55]. A meta-analysis comparing drug prophylaxis and GCS in craniotomy patients showed that drug administration was superior to GCS alone [2]. However, the administration of heparin is associated with a considerable risk of bleeding during intracranial surgery, so that it is generally not used during the procedure but is started after several hours [11, 44, 47]. Interestingly, other studies have shown that the initiation of drug prophylaxis after surgery has no effect on the incidence of VTE [50], and on the other hand, the length of time until the start of prophylaxis correlates with the risk of VTE [14]. This means that intraoperative prophylaxis is especially important.

In addition to GCS, IPC can be used intraoperatively for mechanical prophylaxis. In a small pilot study of our department, this method was successful in reducing the incidence of VTE from 26.4% to 7.3% [38]. In this much larger cohort here, the incidence of DVT was also significantly lower in the group with intraoperative IPC and D-dimer evaluation (4.4%) than in the group with D-dimer evaluation alone (22.6%) (*p* < 0.001). The incidence of PE also decreased to 0.8% compared to 5.1% (*p* < 0.001). However, the type of thrombosis (classic DVT, muscle vein, CVC-associated) did not seem to be influenced by IPC. In regression analysis, not using IPC was highly significantly associated with the occurrence of VTE (*p* = 0.004).

A prospective randomized study of the use of IPC in elective craniotomy cases showed similar results, with a reduction in VTE from 22.9% to 9.6% [39]. IPC has also been beneficial in reducing VTE rates in other procedures. For example, a meta-analysis showed the substantial advantage of IPC in combination with GCS in gynecological procedures [26]. The benefit of IPC has also been demonstrated for surgery in patients with bronchial carcinoma [54]. The intraoperative use of IPC has also been included in the European Anesthesia Guidelines, although the lack of prospective randomized data is noted here. The use of intraoperative IPC should be considered, especially in patients with an increased risk of bleeding [11].

A potential risk factor for VTE, although controversial, is the underlying tumor entity in craniotomy patients. In addition, a tumor as a cause of craniotomy appears to be associated with a higher risk of VTE compared to vascular disease [4, 22]. In contrast, a different study reported that the underlying disease as the reason for the craniotomy, such as tumor or trauma, made no difference in terms of VTE risk [14]. Other data showed that the tumor entity, whether benign or malignant, did not appear to play a role in VTE risk [4, 5].

In a large study of more than 1500 patients, Shi et al. showed that glial tumors and craniopharyngiomas have an increased risk of VTE [48]. In contrast, Smith et al. reported in another large population of over 1000 patients that the tumor entity did not affect VTE risk [50]. There are also data in the literature suggesting that the diagnosis of meningioma is associated with the highest risk of VTE [45, 53]. Conversly, a meta-analysis found that high-grade glioma is a risk factor for VTE [57]. This observation is consistent with the data from our regression analysis. This showed that WHO grade 4 tumors were an individual risk factor. In addition, there is some data to suggest that IDH (Isocitrate dehydrogenase) status may play a role in this regard [9]. However, this factor was not taken into account in our analysis, so we cannot draw any conclusions based on our data. Regression analysis also showed that patients with cerebral metastases appeared to have the highest risk of VTE (p < 0.0001). The factor cerebral metastasis was rarely evaluated, although one study supports this assumption and shows similar results, which however were not significant here [43]. On the other hand, the study by Kaewborisutsakul et al. reports that metastases do not increase the risk of VTE [18].

Another possible factor influencing the risk of VTE is patient positioning during surgery. Data on this are limited in the literature. Gessler et al. found that the seated position was a predictor of the development of VTE [12]. But other studies revealed that positioning is not a risk factor [50]. In our study, we also found that patient positioning during surgery had no significant effect on the development of VTE.

There is also little data in the literature on other factors such as gender or age and the risk of VTE. Some studies have shown an increasing risk after 60 years of age [22, 47–49]. At least a trend can be inferred from our data, although this factor was not significant in the regression analysis (p = 0.072). Regarding gender, there is some data in which men were more frequently associated with VTE [47]. In opposite, another study found no effect of gender [14]. This is consistent with our data, where we also found no difference between VTE risk and gender.

## Limitations

This is a retrospective data analysis, which implies that the data quality is somewhat limited, especially for cases that occurred a long time ago (without digital medical records).

## Conclusion

The intraoperative use of IPC cuffs during craniotomy in patients with brain tumors should be standard practice, as it significantly reduces the risk of perioperative development of VTE. In addition, D-dimer should be measured on the third postoperative day, as many DVTs, even asymptomatic ones, can be detected in this way. Early detection allows appropriate treatment to be initiated, thereby reducing the risk of fatal PE.

## Data Availability

The complete data can be made available on request from the author.
